# Evaluation of CD10 expression in prostate adenocarcinoma and its clinic-pathological correlation at tertiary care hospital

**DOI:** 10.6026/973206300223380

**Published:** 2026-06-30

**Authors:** Hukam Singh Meena, Radhika Rai, Aashita Thakur, Shashi Kant, Ashok Panchonia, Meena Mittal, Chandrashekhar Chhatrasal

**Affiliations:** 1 Department of Pathology, Mahatma Gandhi Memorial Medical College, Indore, Madhya Pradesh, India

**Keywords:** Prostate cancer, PSA, gleason score, prognostic factors

## Abstract

Prostate adenocarcinoma is the second most common cancer in men worldwide and ranks sixth in cancer-related mortality, with more than 95% of cases arising from prostatic acini. CD10, an immunohistochemical marker, has emerged as a potential prognostic indicator, as its high expression is linked to aggressive tumor behavior and early lymph node metastasis, and tumor grading is performed using the WHO Grade Group system based on the Gleason score. Hence, this study included 40 cases of prostate adenocarcinoma diagnosed between January 2023 and December 2024 at the Department of Pathology, MGM Medical College, Indore, involving patients aged 45-95 years with a mean age of 68.5 years. Among the cases evaluated, CD10 expression was negative in 30% (n=12), diffuse in 27.5% (n=11), cytoplasmic in 22.5% (n=9), and membranous in 20% (n=8), and these patterns were correlated with Gleason score. Thus, we show that the pattern of CD10 immunoexpression, particularly diffuse and cytoplasmic positivity, correlates significantly with higher Gleason scores in prostate adenocarcinoma, supporting its incorporation as an adjunct immunohistochemical marker to refine histopathological risk stratification beyond conventional Gleason grading.

## Background:

Adenocarcinoma of the prostate is the second most common cause of cancer and sixth leading cause of cancer-related deaths in men worldwide. Incidences in India - The incidence rate of prostate cancer are estimated to be 9 per 100,000 men in the whole of India. Over 95% of prostatic cancers are adenocarcinoma that arises in prostate acini [1]. Clinically prostate cancer may be asymptomatic and its natural progression is relatively slow. Usually it is detected by suspicious nodule on digital rectal examination (DRE) or raised prostate specific antigen (PSA) levels. Urinary symptoms such as hesitancy, dysuria, increased frequency or haematuria occur and are seen in advanced cases [2]. Prostate specific antigen is clinically useful biochemical marker in the prostate cancer. However PSA is not cancer specific as serum PSA levels get elevated in various non-malignant conditions such as BPH, prostatitis and certain medical procedures. Some prostate cancers, particularly early stage cancer do not produce significant PSA elevation leading to misdiagnosis [3]. The new biomarkers have the potential to provide an opportunity to better define groups of men at high risk of developing prostate cancer, to improve the screening techniques and to discriminate indolent versus aggressive disease. Now a day, immunohistochemistry markers are used for prostate cancers, among which CD10 marker has a prognostic role. CD10 is a transmembrane metallo-endopeptidase that cleaves and inactivates a variety of peptide growth factors [4]. Loss of CD10 expression is a common, early event in human prostate cancer. Prostate tumors expressing high levels of CD10 has a more aggressive biology with an early propensity towards lymph node metastasis. CD10 plays an important role in the pathogenesis of prostate cancer. The loss of CD10 is a common early event in human prostate cancer and is seen in lower Gleason Score malignancies while increased and altered expression is seen in high Gleason Score tumours, lymph nodes and bone metastasis [5]. The histological grading was done by WHO Grade Group system based on Gleason Score. Cases having Gleason Score ≥ 6 were considered as malignant. Correlation between Gleason score and CD 10 marker may serve as important prognostic marker in prostate cancer. Therefore, it is of interest to evaluate the expression of CD10 in adenocarcinoma to assess the severity of the disease and determine the expression of IHC marker CD10 in prostate adenocarcinoma.

## Materials and Methods:

The study was carried out at the Department of Pathology, MGM Medical College Indore (M.P.) from January 2023 to December 2024 (two year duration). The study included 40 cases. Patients presented to Urology OPD and on examination having increase PSA (4ng/mg) and hard nodule on DRE (suspicious of prostate carcinoma) planned for prostate needle biopsy and reported positive. The patients were in age group of 45-95 years with a mean age of 68.5 years. Patient operated at urology department Supers Speciality Hospital Indore insidiously detected carcinoma prostate after TURP during histopathological examination. Inclusion and exclusion criteria have been applied to the patients and cases have been selected fulfilling the criteria. Consent of the patients has been taken. Then, paraffin embedded blocks of the tissue has been taken for immunohistochemistry study. The CD 10 expression was assessed and correlated with Gleason score/grade of reporting prostate carcinoma.

## Intensity of staining:

0: Negative

1: focally positive

2: diffusely positive

## Pattern of CD10 expression:

[1] Membranous

[2] Cytoplasmic

1) Staining in <5% of tumor cells was considered as negative.

2) Staining in 5-20% of tumor cells - focally positive.

3) Staining in >20% of tumor cells - diffusely positive.

The histopathological diagnosis was established on routine H & E staining.

The histological grading was done by WHO Grade Group system based on Gleason Score. The cases were categorized according to

Gleason Score and WHO Grade Group system as follows:

[1] Grade Group I (Gleason Score ≤ 6) - Only individual discrete well-formed glands.

[2] Grade Group II (Gleason Score 3+4=7) - Predominantly well-formed glands with a lesser component of poorly-formed/fused/cribriform glands.

[3] Grade Group III (Gleason Score 4+3=7) - Predominantly poorly-formed/fused/cribriform glands with lesser component of well-formed glands.

[4] Grade Group IV (Gleason Score 8)

[5] Only poorly-formed/fused/cribriform glands or

[6] Predominantly well-formed glands and lesser component lacking glands or

[7] Predominantly lacking glands and lesser component of well-formed glands.

[8] Grade Group V (Gleason Score 9-10) - Lack of gland formation (or with necrosis) with or without poorly-formed/fused/cribriform glands [4].

## Ethical consideration:

This study was approved by institutional ethics committee

## Statistical analysis:

The results have been analysed using various tables. Data were analysed by using statistical software SSPS version 25. Correlation accessed via Spearman's rank and Pearson's coefficient; statistical significance (p<0.05) determined.

## Results:

Overall, the total number of patients included in the study was 40. The majority of the patients in the study belonged to the age group of 70-79 years, comprising 50% (n=20) followed by 22.5% (n=9) in the 60-69 years age group and 15% (n=6) in the 50-59 years age group. Patients aged above 80 years accounted for 10% (n=4), while the smallest proportion, 2.5% (n=1), was in the 40-49 years age group. The most commonly reported complaint among the study participants was retention of urine, noted in 24.4% (n=11) of cases, followed by obstructive voiding, reported by 17.8% (n=8) of participants. Complaints such as dysuria, difficulty in micturition, poor urine stream, and lower back ache were each observed in 11.1% (n=5) of participants. Nocturnal polyuria and hematuria were the least frequently reported complaints, each comprising 6.7% (n=3) of the total. CD10 expression in more than 20% of cells was observed in 40% (n=16) of the cases, making it the most common category. An equal proportion of participants (30%, n=12 each) demonstrated CD10 expression in less than 5% of cells and between 5-20% of cells, respectively (Table 1). The most frequently observed pattern was negative expression, noted in 30% (n=12) of the cases, followed by diffuse expression in 27.5% (n=11), cytoplasmic expression in 22.5% (n=9), and membranous expression in 20% (n=8) of the participants (Table 2). A statistically significant correlation was observed between CD10 expression patterns and Gleason score (p = 0.003). Among participants with a Gleason score of 6, CD10 expression was entirely negative. In those with a Gleason score of 7, the most frequent pattern was negative expression (55.6%), followed by membranous (33.3%) and cytoplasmic (11.1%) expression. Participants with a Gleason score of 8 exhibited a more diverse expression pattern: diffuse (37.5%), cytoplasmic (25%), membranous (25%), and negative (12.5%). In contrast, those with a Gleason score of 9 predominantly showed diffuse (58.3%) and cytoplasmic (41.7%) expression, with no cases showing membranous or negative staining. These findings suggest that higher Gleason scores were more frequently associated with diffuse and cytoplasmic patterns of CD10 expression, while lower scores tended to show negative or membranous patterns (Table 3). The correlation between CD10 expression patterns and PSA levels was not statistically significant (p = 0.55). Participants with PSA levels <50 ng/mL and 50-100 ng/mL each accounted for one case (2.5%) and showed exclusively negative CD10 expression. Among the majority of participants (n=38) who had PSA levels >100 ng/mL, the distribution of CD10 expression patterns was more varied. Diffuse expression was observed in 28.9%, cytoplasmic in 23.7%, membranous in 21.1%, and negative expression in 26.3% of these cases. Although a diverse expression pattern was noted in the group with PSA >100 ng/mL, no clear association was found between PSA levels and specific CD10 expression patterns based on the statistical analysis (Table 4). These findings suggest that higher Gleason scores were more frequently associated with diffuse and cytoplasmic patterns of CD10 expression, while lower scores tended to show negative or membranous patterns (Figure 1, Figure 2 - see PDF).

## Discussion:

Prostate adenocarcinoma remains one of the most prevalent malignancies in males, particularly in older individuals. This study investigated the expression pattern of CD10 - a transmembrane metallo-endopeptidase - in prostate adenocarcinoma and attempted to correlate its immunohistochemical expression with clinicopathological parameters including Gleason score. The mean age group most affected was 70-79 years (50%), consistent with previous literature Wasai *et al.* [5] and Kratzer *et al.* [6] indicating increasing incidence of prostate cancer with the age. Clinical symptoms were non-specific, with urinary retention and obstructive voiding being the most common presenting complaints. This study correlated with Alizadeh *et al.* [7] which stated most common presenting symptom to be urinary irritative and obstructive symptoms. In terms of CD10 expression, 40% of cases demonstrated expression in more than 20% of tumor cells, 30% of cases demonstrated expression in 5-20% of tumor cells, and 30% of cases showed no CD10 expression. A significant correlation was found between CD10 expression pattern and Gleason score (p=0.003). High grade tumors (Gleason 9 and 10) predominantly showed diffuse and cytoplasmic CD10 expression, whereas low-grade tumors (Gleason 6 and 7) showed either negative or membranous expression. This aligns with published studies, including Van Leenders *et al.* [8], Tawfic *et al.* [9], Era *et al.* [10] and Albrecht *et al.* [11], noted increased CD10 expression correlates with tumor grade and aggressiveness. A study done by Kaur *et al.* [12] also reported that CD10 expression increases with advancing Gleason grade and can be used for tumor stratification.

## Conclusion:

CD10 expression, particularly diffuse and cytoplasmic patterns, is significantly associated with higher Gleason scores and more aggressive prostate cancer behavior. No significant correlation was observed between CD10 expression and PSA levels. Data shows the potential role of CD10 as an adjunctive prognostic biomarker for risk stratification in prostate cancer.

## Figures and Tables

**Table 1:** Distribution of CD10 Expression (Immunohistochemistry) among study patients

**IHC (CD10) % of cells**	**N**	**%**
<5	12	30
20-May	12	30
>20	16	40
Total	40	100

**Table 2:** Pattern of CD10 Expression among study participants

**Pattern of CD10 expression**	**N**	**%**
Cytoplasmic	9	22.5
Diffuse	11	27.5
Membranous	8	20
Negative	12	30
Total	40	100

**Table 3:** Correlation of CD10 Expression (%) with Gleason Score

**Gleason score**		**Pattern of CD10 expression**				**Total**	**Chi-square test**
		Cytoplasmic	Diffuse	Membranous	Negative		p-Value
6	N	0	0	0	1	1	0.003**
	%	0.00%	0.00%	0.00%	100.00%	100.00%	
7	N	2	0	6	10	18	
	%	11.10%	0.00%	33.30%	55.60%	100.00%	
8	N	2	3	2	1	8	
	%	25.00%	37.50%	25.00%	12.50%	100.00%	
9	N	5	7	0	0	12	
	%	41.70%	58.30%	0.00%	0.00%	100.00%	
10	N	0	1	0	0	1	
	%	0.00%	100.00%	0.00%	0.00%	100.00%	
Total	N	9	11	8	12	40	
	%	22.50%	27.50%	20.00%	30.00%	100.00%	

**Table 4:** Correlation of CD10 Expression (%) with PSA Level among study participants

**PSA level (ng/ml)**		**Pattern of CD10 expression**				**Total**	**Chi-square test**
		Cytoplasmic	Diffuse	Membranous	Negative		p-Value
<50	n	0	0	0	1	1	0.55
	%	0.00%	0.00%	0.00%	100.00%	100.00%	
50-100	n	0	0	0	1	1	
	%	0.00%	0.00%	0.00%	100.00%	100.00%	
>100	n	9	11	8	10	38	
	%	23.70%	28.90%	21.10%	26.30%	100.00%	
Total	n	9	11	8	12	40	
	%	22.50%	27.50%	20.00%	30.00%	100.00%	
